# Congenital collagenopathies increased the risk of inguinal hernia developing and repair: analysis from a nationwide population-based cohort study

**DOI:** 10.1038/s41598-022-06367-5

**Published:** 2022-02-11

**Authors:** Hao-Han Chang, Yung-Shun Juan, Ching-Chia Li, Hsiang-Ying Lee, Jian-Han Chen

**Affiliations:** 1 Department of Urology, Kaohsiung Medical University Hospital, No. 100, Tzyou 1st Rd., Sanmin Dist., Kaohsiung, 80756 Taiwan; 2 Department of Urology, School of Medicine, College of Medicine, Kaohsiung Medical University, Kaohsiung, Taiwan; 3 Urology Department, Kaohsiung Municipal Ta-Tung Hospital, Kaohsiung, Taiwan; 4 Graduate Institute of Clinical Medicine, College of Medicine, Kaohsiung Medical University, Kaohsiung, Taiwan; 5 Department of Bariatric and Metabolic International Surgery Center, E-Da Hospital, No. 1, Yida Rd., Yanchao Dist., Kaohsiung, 82445 Taiwan; 6 Division of General Surgery, E-Da Hospital, Kaohsiung, Taiwan; 7 School of Medicine, College of Medicine, I-Shou University, Kaohsiung, Taiwan

**Keywords:** Clinical genetics, Medical genetics

## Abstract

Herein, we aimed to explore whether male patients with congenital collagen diseases had a higher risk of inguinal herniation than patients without these diseases. Data were retrospectively collected from the National Health Insurance Research Database of Taiwan. The study cohort included 1,801 male patients diagnosed with congenital collagen diseases based on the ICD-9 CM diagnostic codes; after propensity score matching, the control group comprised 6,493 men without congenital collagen diseases. The primary endpoint was inguinal hernia repair during the observation period. During a median follow-up period of 133.9 months, the risk of inguinal herniation in the collagen group was significantly higher than that in the control group (HR = 2.237, 95% CI 1.646–3.291, p < 0.001). This phenomenon was observed in patients younger than 18 years (HR: 3.040, 95% CI 1.819–5.083, p < 0.001) and in those aged 18–80 years (HR: 1.909, 95% CI 1.186–3.073, p < 0.001). Asian men with congenital collagen diseases are at a high risk of developing inguinal hernias, regardless of age. Detailed physical examination and patient education should be performed for these patients to prevent inguinal herniation.

## Introduction

Inguinal hernia repair, executed over 20 million times annually, is one of the most common surgical procedures worldwide^1,2^. In recent years, the number of surgical techniques for treating inguinal hernias has increased. Currently, in addition to the traditional open approach, laparoscopic approaches, including the transperitoneal, extraperitoneal, or single port approaches are accepted and executed by surgeons. A previous study reported that the laparoscopic approach was associated with a lower recurrence rate than that of the open approach without mesh repair^3^. Single-incision laparoscopic extraperitoneal repair procedures may be more advantageous than the those where the open approach is used, owing to decreased pain and better cosmetic outcomes associated with the laparoscopic approach^4^. New techniques, including the microsurgical assist^5^ and transinguinal peritoneal approaches^6,7^, are now used along with traditional open hernia repair approaches. Thus, there is an ongoing debate regarding the necessity of regular microscopic examination of the hernia sac.

Traditionally, the etiology of an inguinal hernia can be congenital or acquired. The congenital type is caused by a patent processus vaginalis, which is an invagination site in the peritoneum that should have closed during embryo development^8^. It is the most frequently occurring pediatric hernia type; it is also associated with a high requirement for secondary repair^9^. Acquired hernias are caused by weakening or dehiscence of the fascial structure accompanied by loss of abdominal wall strength, which allows the hernia sac to drop out easily^10^. Factors such as mechanical strain, previous operation, and intra-abdominal pressure also contribute to hernia formation^11^. If these risk factors persist after initial inguinal hernia repair, the patient has a higher risk of contralateral inguinal hernia development^12^.

Recent studies have focused on the biology of the cause of hernia. Collagen, the main protein that forms the extracellular matrix (ECM), has been extensively studied. Collagens are a large family of proteins that are important for tissue scaffolding, cell adhesion, cell migration, and tissue repair^13^. While collagen can be classified into several types, disorders of type I, III, IV, and V collagen distribution are reportedly related to hernia formation. Type I collagen is related to the strength of the fascia or mature scar, while type III collagen, which is synthesized during early wound healing, is unstable and is characterized by reduced cross-linking^14^. When tissue injury occurs, fibroblasts initially gather at the injury site and preferentially produce type III collagen^15^. A decreased amount of type I collagen and increased amount of type III collagen lowers the type I to type III collagen ratio, reduces tensile strength, and may contribute to hernia formation^14,16,17^. In addition, a small increase in type III collagen gene expression may eventually cause hernia formation^18^. Type V collagen, which is important for fibrilogenesis, may also be an important contributor to the development of an inguinal hernia. In a previous study, the turnover rate of type V collagen was observed to be persistently altered in patients with inguinal herniation^19^. Furthermore, patients with an inguinal or incisional hernia were found to have an increased turnover of type IV collagen^20^.

There are several types of congenital connective tissue diseases that cause collagenopathies, including Ehlers–Danlos syndrome (EDS), osteogenesis imperfecta, chondrodystrophy, and osteodystrophies. We hypothesized that patients with congenital connective tissue disorders have quantitative or qualitative defects in the collagen; these defects are strongly related to inguinal hernia formation. Several published case reports have demonstrated an association between connective tissue disease and all hernia types^21–23^; however, to the best of our knowledge, high-level clinical evidence linking collagenopathy and inguinal herniation is lacking. In this study, we investigated the relationship between congenital collagenopathy and inguinal herniation by conducting a nationwide population-based cohort study in Taiwan.

## Methods

### Database

We retrospectively conducted a national cohort study using the National Health Insurance Research Database (NHIRD) of Taiwan, which is regulated and maintained by the Data Science Centre of the Ministry of Health and Welfare of Taiwan. The NHIRD is the database of National Health Insurance (NHI) program in Taiwan, which includes over 23 million Taiwanese people, almost all of whom reside in Taiwan. Thus, the database can be regarded as containing medical records of the entire Taiwanese population. In this database, the clinical diagnosis of the patients was determined according to the International Classification of Diseases, 9th revision, Clinical Modification (ICD-9-CM). This study was approved by the Institutional Review Board of the E-Da Hospital (EMRP-106-063) and Kaohsiung Medical University Hospital (KMUHIRB-E(I)-20180308), and was conducted in accordance with the Declaration of Helsinki. The requirement for informed consent for study participation was waived by the Institutional Review Boards of E-Da Hospital (EMRP-106-063) due to the retrospective case control design of the study.

### Inclusion and exclusion criteria

The study participants were selected using NHIRD data for the period from January 1, 2003, to December 31, 2013. Patients diagnosed with congenital collagen diseases were included in the collagen group. Diagnoses were established based on ICD-9-CM codes 756.51, 756.59, 756.83, 756.89, and 756.4, which represent osteogenesis imperfecta, Albright syndrome, EDS, amyotrophia congenita, and chondrodystrophy, respectively. The index day was January 1, 2013. We excluded female patients, patients who were born after the index day, and patients who died before or underwent hernia repair before the index day.

Further, we randomly sampled 50,000 active civilians to be included in the general population group. After excluding those included in our experimental cohort and those who underwent hernia repair before the index day, patients were chosen based on a propensity score-matched (1:4) analysis that included age, the Charlson Comorbidity Index (CCI) score, and comorbidities such as chronic obstructive pulmonary disease (COPD), prostate disease, or obesity.

**Figure 1:**
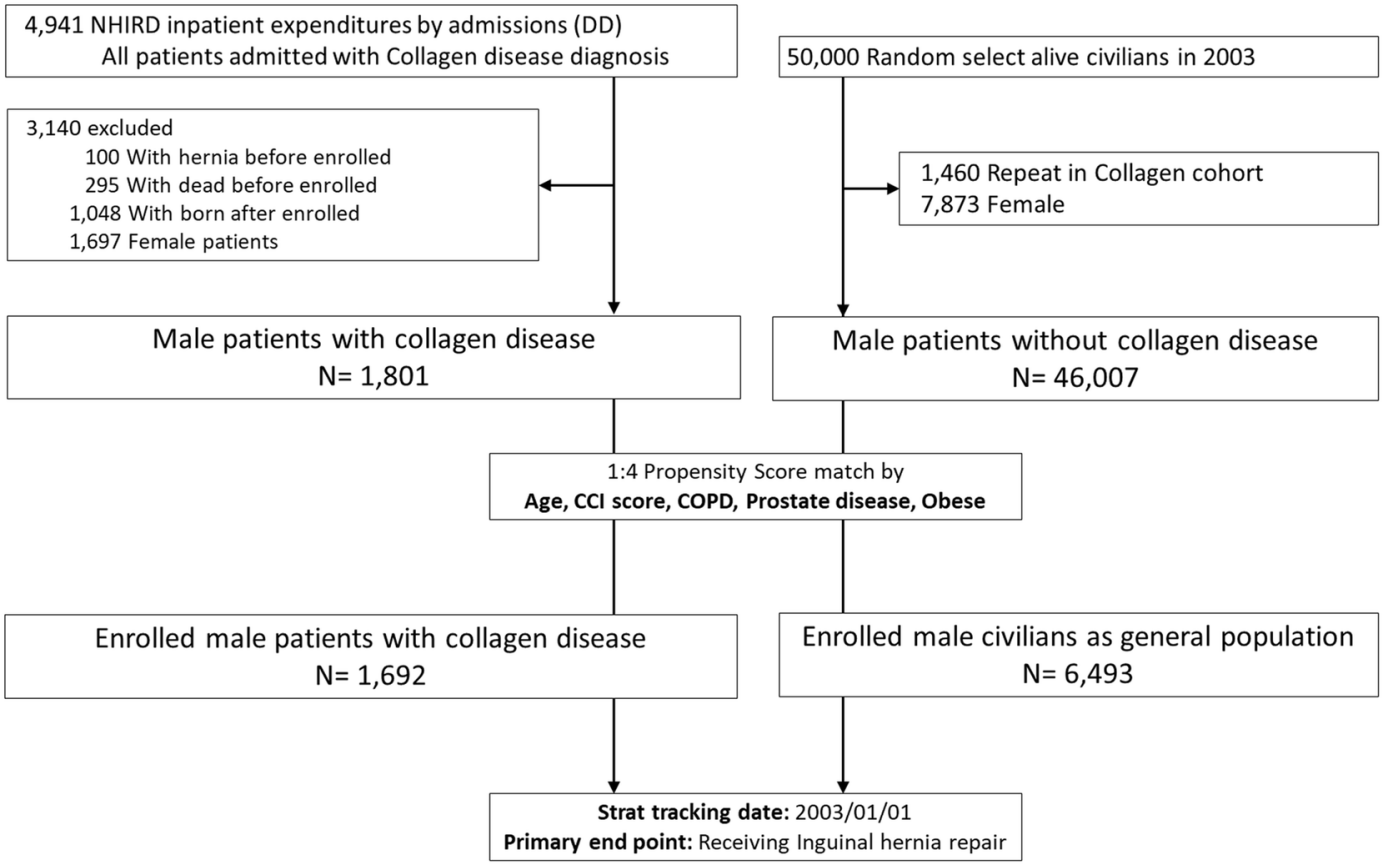
Flow chart showing the inclusion and exclusion criteria of this study.

All included patients were followed up until their withdrawal record presented in the NHI or the end of our study period, December 31, 2013. A flowchart of the selection criteria is shown in Fig. 1.

### Study outcomes and covariates

The primary outcome of our study was inguinal hernia repair during the observation period. The diagnosis of hernia was confirmed using the ICD-9 CM codes for hernia (550.xx to 553.xx) and for surgical procedures that included inguinal hernia (53.00–53.05)^12,24,25^. In Taiwan, the costs of hernia repair, including both the traditional open and laparoscopic assisted approaches, were covered by the National Health Insurance. Thus, all medical records had been evaluated thoroughly to ensure that the diagnosis and treatment were appropriate. The follow-up time was defined as the duration from the beginning of inclusion in the study until the patient underwent hernia repair or until the end of the study.

In our study, patients’ demographic data, including age and baseline comorbidities, were recorded. Baseline comorbidities that were reported to be potential risk factors of hernia in prior studies and those that may have affected our results were examined. During the analysis, we assessed several independent variables as comorbidities, including prostate disease (ICD-9-CM codes 600.x, 601.x, 602.x)^12^, obesity (ICD-9-CM code: 278.00, 278.01)^26^ and COPD (ICD-9-CM code: 491.x–496.x, 501.x–504.x), which was reported as a risk factor for hernia repair^12,27^. Comorbidities identified by an ICD-9 code in the NHIRD database prior to admission were also evaluated.

### Statistical analysis

The baseline characteristics of the two groups (congenital collagen disease cohort and the general population) were analyzed using descriptive statistics. A Kaplan–Meier curve was used to estimate the cumulative incidence of hernia repair in the two groups, and the differences between the two groups was analyzed using the log-rank test. Hazard ratios (HRs) with 95% confidence intervals (CIs) were calculated using the chi-square test, and Cox proportional hazards models adjusted for multiple variables were used to test the association between the two groups. Statistical analyses were conducted using the SPSS version 25 software (IBM, Chicago, IL, USA). Statistical significance was set at p < 0.05.

## Results

Between January 1, 2003, and December 31, 2013, we identified 4941 patients who were diagnosed with congenital collagen disease based on ICD-9-CM coding. Of these, 3140 patients met one or more of the exclusion criteria (Fig. 1); 100 patients were excluded due to a past history of hernia surgery, 295 patients had died before the enrollment period, 1048 patients were born after the index day, and 1,697 patients were excluded as they were female. Finally, 1692 male patients with congenital collagen diseases were included in the collagen group. Of the 50,000 civilians that were screened, 1460 were excluded because they were already included in the collagen group and 7873 patients were excluded due to female sex. Of the remaining 46,007 patients, propensity score matching based on age and comorbidities was performed for the collagen group at a ratio of 1:4. In total, 6,493 male civilians were enrolled in the general population (control) group. The algorithm for the division of patients and civilians according to the inclusion and exclusion criteria is shown in Fig. 1.

Table 1 presents the baseline characteristics of the collagen and control groups. There were no significant between-group differences in age and comorbidities related to inguinal herniation, including COPD^28^, prostate disease^29^, and obesity^30^. The median age of patients in the control and collagen groups was 13.41 (interquartile range, 5.50–30.50) and 13.42 (interquartile range, 5.35–30.47), respectively. As connective tissue disease was a contributing factor for the Charlson Comorbidity Index (CCI) score, the CCI score of the collagen group was significantly higher than that of the control group (p < 0.001).

**Table 1:** Basic characteristics of the collagen diseases group and control group in Asian adult male population.

Variables	Control group (N = 6493)		Collagen group (N = 1692)		P value
	Median	IQR	Median	IQR	
Age	13.41	25.00	13.42	25.13	0.749
CCI score	0	0	0	0	< 0.001

Risk factors	N	(%)	N	(%)	
COPD	274	4.22	62	3.66	0.305
Prostate disease	88	1.36	21	1.24	0.715
Obesity	4	0.06	2	0.12	0.444

**Figure 2:**
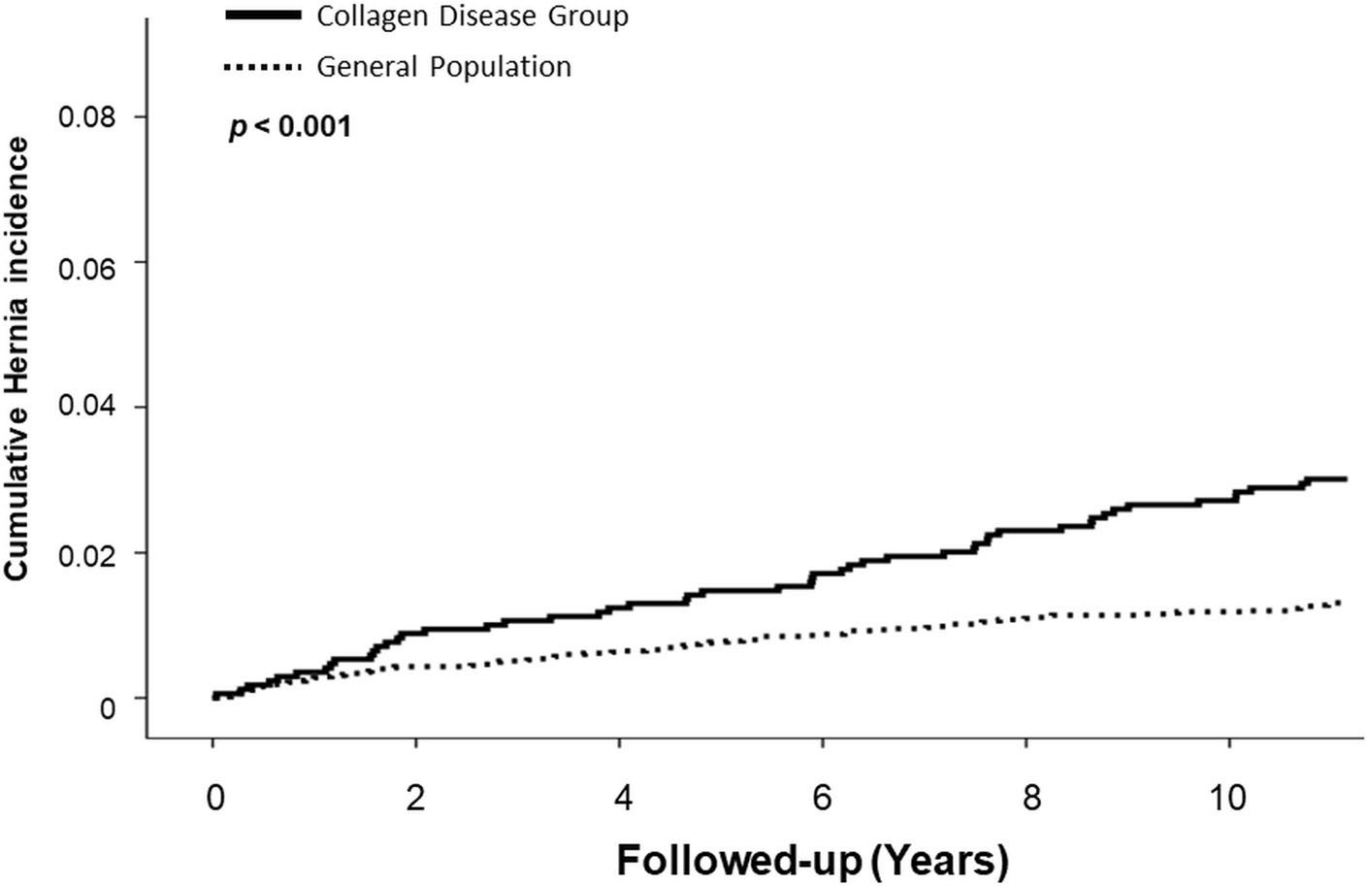
Cumulative incidence of hernia development. The solid line represents the collagen disease group while the dotted line represents the general population.

During a median follow-up period of 132.85 and 131.17 months for the control and collagen groups, respectively, 1.3% and 3.0% of patients underwent hernia repair, respectively. Patients in the collagen group exhibited a significantly increased risk of developing inguinal hernia (HR, 2.237; 95% CI 1.646–3.291; p < 0.001) compared to that in the control group (Table 2). Figure 2 shows the cumulative incidence curves for the cumulative probability of receiving hernia repair for both groups in the propensity score-matched cohort. To determine whether this tendency existed in both adults and children, we divided the patients into two groups based on age, with the cut-off age being 18 years. In patients aged < 18 years, the incidence of inguinal hernia repair was significantly higher in the collagen group (0.9% vs. 2.6%; HR, 3.040; 95% CI 1.819–5.083; p < 0.001). A similar tendency was observed in patients aged > 18 years (2.0% vs. 3.6%; HR, 1.909; 95% CI 1.186–3.073; p = 0.008). The risk of developing both unilateral (1.22% vs. 2.36%; p < 0.001) and bilateral (0.11% vs. 0.59%; p < 0.001) inguinal herniation was significantly higher in the collagen group than in the control group (Table 3). Table 4 presents the results of the multivariate analyses using the Cox regression models. Age and collagen disease appeared to be independent risk factors for the development of an inguinal hernia.

**Table 2:** The risk of collagen diseases group and control group in male adult civilians.

	*No. cases	(%)	HR	95% CI	P value
**All age**					
Control group	86	(1.3%)	Ref		
Collagen group	51	(3.0%)	2.237	1.646–3.291	< 0.001
**Age < 18**					
Control group	33	(0.9%)	Ref		
Collagen group	26	(2.6%)	3.040	1.819–5.083	< 0.001
**Age = 18 ~ 80**					
Control group	53	(2.0%)	Ref		
Collagen group	25	(3.6%)	1.909	1.186–3.073	0.008

*Statistical significance was set at p < 0.05

**Table 3:** The incidence of developing unilateral, bilateral and all hernia between collagen group and control groups.

	Collagen disease (%)	Control group (%)	P value
All Hernia	3.01	1.32	< 0.001
Unilateral	2.36	1.22	< 0.001
Bilateral	0.59	0.11	< 0.001

**Table 4:** Multivariable analysis for risk of hernia among patients over 18 years old.

	HR	95% CI	P value
Collagen disease	1.907	1.185–3.070	> 0.01
Age	1.037	1.025–1.049	> 0.01
COPD	0.523	0.157–1.725	0.287
Prostate disease	1.162	0.509–2.653	0.722
Obesity	0.001	0.000–0.001	0.965

## Discussion

The etiology of inguinal hernia development is complex and multifactorial. Recently, studies have focused on biological and genetic factors for hernia development, including the components of the extracellular matrix, the amount and ratio of different types of collagen, and genetic variants of the related genes. Collagen, which affects the elasticity and resistance of the transversalis fascia, can be classified into more than 30 types^31^. The various types of collagen have different characteristics and functions. Type I collagen, which is the most abundant form of collagen throughout the body^32^, is considered to be responsible for the strength and mechanical durability of the tendons^33^. During the healing process, a large amount of type III collagen is formed at the wound site. The fibrils of type III collagen are thinner than those of type I collagen^34^. Decreasing the ratio of type I to type III collagen has been shown to decrease the strength and elasticity of the tendon and fascia, thereby increasing the possibility of inguinal hernia formation^16,17^. Certain congenital connective tissue diseases cause collagenopathy, i.e., quantitative or qualitative defects in the collagen. However, to the best of our knowledge, the direct relationship between congenital collagen or connective tissue diseases and the risk of inguinal hernia formation has rarely been reported. Herein, we conducted a nationwide population-based cohort study in Taiwan to investigate the relationship between congenital collagen diseases and inguinal hernias.

In this propensity score-matched cohort, we reported that male patients with congenital collagenopathy had a high rate of inguinal herniation development. We observed that the incidence of inguinal hernia was 2.237 times higher in male patients who were diagnosed with congenital collagen disease than in the general population. (HR: 2.237, 95% CI 1.646–3.291, p < 0.001). A multivariate analysis was performed to examine the effects of multiple independent variables on hernia development. Our study showed that age was an independent risk factor for hernia development. To diminish the effect of age, we divided the patients into two groups: those aged under and over 18 years. The risk of developing inguinal hernia was significantly higher in the collagen group in both age groups; the HR was 3.040 in patients aged < 18 years (95% CI 1.819–5.083, p < 0.001) and 1.909 in patients aged 18–80 years (95% CI 1.186–3.073, p < 0.001).

Several possible mechanisms can explain this phenomenon. In most types of congenital collagenopathies, including EDS, osteogenesis imperfecta, chondrodystrophy, and osteodystrophies, the major pathophysiologic factor is alterations in the genes involved in collagen synthesis and processing of different types of collagen^35–37^. EDS is an inherited connective tissue disorder characterized by defects in collagen synthesis, causing progressive deterioration of collagens. The clinical presentation of this condition includes soft skin, skin fragility, delayed wound healing, easy bruising, and joint hypermobility^38^. EDS can be classified into more than 10 different subtypes based on the particular defect in collagen metabolism and gene mutation. In 2017, an updated International Classification of EDS identified 13 variants of the condition, with mutations in 19 distinct genes^39^. These mutations cause molecular or biochemical defects in collagen types I, III, and V and/or the related enzymes^40^. Osteogenesis imperfecta, which is the most common cause of congenital bone fragility, is a disease that leads to defects in type 1 collagen. In addition to well-known mutations in the *COL1A1* and *COL1A2* genes, several other proteins are reportedly involved in the pathogenesis of this disease^41^. The total amount of collagen may be related to herniation development. Antonio Britto Casanova et al. reported that the total collagen amount was 17.3% lower in patients with hernias than in the healthy controls. This decreasing tendency was more prominently observed for type I collagen than for type III collagen (23.7% vs. 6.4%)^34^. Wagh et al. also suggested that decreasing the amount of collagen in the rectus sheath would lead to inguinal herniation. Therefore, patients with congenital collagen disease, in whom the quality and quantity of collagen is affected, may have a higher risk of developing herniation. Furthermore, a previous study showed that herniation may be caused by an imbalance between the interstitial collagen and the basement membrane^34^. This phenomenon could result from inadequate turnover of types III, IV, and V collagen. Thus, a systemically poor ECM quality and reduced collagen synthesis may be considered signs of hernia formation^42^. Congenital collagen disease leads to a decrease in the quantity and a poorer quality of collagen, which are significantly associated with inguinal hernia formation. This may explain the increased risk of inguinal herniation in patients with congenital collagen diseases.

To the best of our knowledge, our study is unique in that it is the first study to directly report on the risk of inguinal herniation among patients with collagenopathies. The strength of this study is that it is a nationwide, population-based study investigating the relationship between congenital collagenopathy and autoimmune diseases. More than 99% of the total population of Taiwan is covered under the NHI system. Taiwan’s NHIRD is one of the few nationwide databases maintained by an Asian country.

Our study has several limitations. First, this was a non-randomized analysis registered in the NHI database. We attempted to decrease the selection bias and cohort heterogeneity by using propensity score matching. However, selection bias to a certain degree remained inevitable. Second, data were extracted from the NHI database using the ICD-9-CM coding system instead of medical records, and misinterpretation of some data or misclassification of some diagnoses may become a source of potential bias if the coding system has not been well validated. However, the inclusion of the surgical procedure codes may have improved the accuracy of our results, and only patients who underwent surgery have surgical procedure codes. Third, as this was a retrospective study, further prospective studies are required for a deeper understanding of the relationship between collagen disease and inguinal herniation.

In conclusion, this study demonstrated that Taiwanese men with congenital collagen diseases are at a higher risk of inguinal herniation than those without collagen diseases. Clinicians should inform high-risk patients of the possibility of inguinal herniation, and educate them regarding the signs and symptoms of incarcerated and strangulated hernias, which are associated with severe morbidity.

## Data Availability

All data generated or analysed during this study are included in this published article.
